# Loss of *Myostatin* Shapes the Transcriptomic and Epigenetic Landscapes Across Multiple Muscle Types in Cattle

**DOI:** 10.3390/cimb47060431

**Published:** 2025-06-07

**Authors:** Chao Hai, Xuefei Liu, Chunling Bai, Guanghua Su, Lei Yang, Guangpeng Li

**Affiliations:** State Key Laboratory of Reproductive Regulation and Breeding of Grassland Livestock, College of Life Science, Inner Mongolia University, Hohhot 010070, China; h15248037201@163.com (C.H.); liuxuefei1006@126.com (X.L.); chunling1980_0@163.com (C.B.); suguanghua0707@163.com (G.S.)

**Keywords:** *Myostatin* gene editing, meat quality traits, DNA methylation, axon guidance pathway

## Abstract

*Myostatin* (*MSTN*) is a critical regulator of muscle development. This study aimed to investigate the transcriptional and epigenetic mechanisms by which *MSTN* gene editing affects skeletal, cardiac, and smooth muscle function in cattle. The results showed that the *MSTN* gene-edited (MT) cattle skeletal muscle exhibited significantly larger myofiber cross-sectional areas (*p* = 0.049), accompanied by reduced shear force (*p* = 0.044), cooking loss rate (*p* = 0.0029), and pH (*p* = 0.014). Transcriptomic and whole-genome bisulfite sequencing (WGBS) revealed distinct expression and methylation patterns across muscle types. Notably, axon guidance signaling was identified as a shared enriched pathway in both transcriptional and CG/CHG/CHH methylation profiles of the gluteus. Further, 102 differentially expressed genes (DEGs) were commonly identified across all three muscle types; their KEGG enrichment included immune-related and cellular interaction pathways (e.g., antigen processing and presentation, and cell adhesion molecules), many of which intersect with axon guidance functions. Core regulators such as *SEMA3A*, *PLXNA1*, and *NTN1* were epigenetically modulated in MT gluteus and heart. These findings suggest that *MSTN* knockout remodels neuromuscular signaling through muscle-type-specific transcriptional and epigenetic reprogramming.

## 1. Introduction

Muscles play a fundamental role in animal physiology, facilitating movement, circulation, and various metabolic functions. Based on their structural and functional properties, animal muscles are categorized into three main types: skeletal muscle, heart muscle, and smooth muscle [1,2]. Among these, skeletal muscle accounts for approximately 40–50% of total body mass in livestock and directly determines meat yield and quality, making it a central target for genetic and nutritional interventions [3,4,5]. Skeletal muscle, responsible for voluntary movements, consists of multinucleated striated fibers controlled by the somatic nervous system. This muscle type exhibits high plasticity in response to genetic and environmental factors. In contrast, heart muscle is an involuntary, striated muscle exclusive to the heart, regulated by intrinsic pacemaker cells and the autonomic nervous system, ensuring continuous rhythmic contractions for blood circulation [6,7,8]. Smooth muscle, found in internal organs such as the digestive tract and blood vessels, is also under involuntary control and plays crucial roles in visceral functions, including peristalsis and vascular regulation [9,10].

Among the key regulators of muscle development and metabolism, myostatin (MSTN), a member of the transforming growth factor-beta (TGF-β) superfamily, acts as a negative regulator of muscle growth [11,12]. *MSTN* gene knockout (*MSTN*-KO) has been shown to promote increased muscle mass in multiple species, including cattle, pigs, and mice [13,14,15]. Mechanistically, *MSTN* inhibition leads to enhanced myoblast proliferation and reduced differentiation during the prenatal period, resulting in increased muscle fiber number (hyperplasia) [16]. Postnatally, *MSTN* suppression promotes satellite cell activation and fusion to existing fibers, contributing to muscle fiber enlargement (hypertrophy) [17]. Recent studies suggest that *MSTN* may be involved in heart muscle function and smooth muscle homeostasis through its regulatory effects on muscle-specific transcription factors and epigenetic modifications [18,19,20]. In addition to its well-characterized role in regulating skeletal muscle mass, *MSTN* also participates in the functional regulation of cardiac and smooth muscle tissues. This study systematically investigates these effects across multiple muscle types using integrative transcriptomic and epigenetic analyses, providing a comprehensive view of *MSTN*’s broader physiological roles. By uncovering distinct gene expression and DNA methylation patterns in heart and smooth muscle following *MSTN* knockout, our findings expand the understanding of *MSTN* as a systemic modulator of muscle function. These findings will enhance our understanding of *MSTN*’s role beyond skeletal muscle growth and contribute to the development of genetically optimized livestock with improved production traits.

## 2. Materials and Methods

### 2.1. Animals

All animal procedures were conducted in accordance with the ethical guidelines of the Committee on the Ethics of Animal Experiments at Inner Mongolia University (Approval number: IMU-CATTLE-2023-059).

The *MSTN* gene-edited (MT) cattle used in this study were generated in our laboratory through precise gene editing based on CRISPR/Cas9 technology, which targeted exon 1 of the bovine *MSTN* gene. Single guide RNAs (sgRNAs) were designed and transfected into bovine fetal fibroblasts (BFFs), and gene-edited positive clones were identified through sequencing. These edited cells were subsequently used as nuclear donors in somatic cell nuclear transfer (SCNT) to produce cloned animals carrying a biallelic knockout of the *MSTN* gene [21]. Compared to naturally double-muscled breeds such as Belgian Blue or Piedmontese, the use of gene-edited cattle enabled us to control for breed-specific genetic backgrounds and isolate the effects of *MSTN* disruption. Moreover, these gene-edited animals were more accessible within our institutional resources and have been previously shown by our group to exhibit muscle phenotypes similar to those observed in naturally occurring *MSTN* mutant cattle, thus representing a reliable and tractable model for studying *MSTN* function.

**Figure 1:**
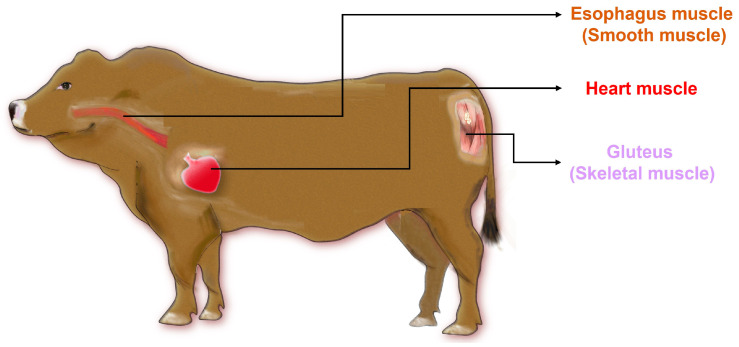
Muscle samples representing all three major muscle types in cattle were collected from distinct anatomical sites. Skeletal muscle samples were obtained from the gluteus muscle, while smooth muscle samples were collected from the esophageal smooth muscle.

To assess the impact of *MSTN* knockout on muscle gene expression and epigenetic modifications, three MT cattle and three age-matched wild-type (WT) cattle from the same breed were selected for analysis. All animals, including MT and WT controls, were Mongolian cattle raised under uniform environmental and management conditions, including consistent feeding and housing practices, to ensure comparability between groups (Supplementary Tables S1 and S2). At approximately 24 months of age, cattle were humanely slaughtered, and samples of three distinct muscle tissues—skeletal muscle from the gluteus medius, cardiac muscle from the left ventricle of the heart, and smooth muscle from the middle segment of the esophagus—were collected. For each tissue type, three replicates (approximately 10 g per sample) were obtained and immediately preserved for subsequent meat quality analysis, as well as transcriptome (RNA-seq) and DNA methylation (whole-genome bisulfite sequencing) analyses (Figure 1). The gluteus muscle was selected as a representative skeletal muscle due to its anatomical consistency and suitability for transcriptomic and epigenetic analyses, offering reliable tissue characteristics. The esophagus was chosen to represent smooth muscle because of its relatively large size and accessibility during dissection.

### 2.2. Hematoxylin and Eosin (HE) Staining of Muscle Tissues

Three types of muscle tissues were collected from each animal within 2 h post-slaughter to ensure tissue integrity. Samples were cut into approximately 0.5 cm × 0.5 cm blocks using sterile scalpels and immediately fixed in 4% paraformaldehyde for 24 h at room temperature. Fixed tissues were then dehydrated through a graded ethanol series (70%, 80%, 95%, and 100%), cleared in xylene, and embedded in paraffin using an automatic tissue processor (Leica TP1020, Leica Biosystems, Wetzlar, Germany). The tissues were sectioned at a thickness of 2–4 μm using a Leica SM 2000 R microtome (Leica Microsystems, Wetzlar, Germany). Sections were then dried overnight at 37 °C.

For histological analysis, paraffin sections were deparaffinized in xylene and rehydrated through a graded ethanol series, and then they were stained with hematoxylin for 5 min, followed by eosin for 2 min, as previously described [22]. After staining, slides were dehydrated through graded alcohols, cleared in xylene, and coverslipped using mounting medium. Images were captured using a light microscope (Nikon Eclipse E100, Tokyo, Japan) equipped with a digital camera.

For histological analysis, transverse sections of gluteus, heart, and esophagus muscles were stained with HE and imaged under a light microscope (Nikon Eclipse E100, Japan). Digital images were analyzed using ImageJ software (v1.53t, NIH, Bethesda, MD, USA). The cross-sectional area of individual muscle fibers was delineated manually using the polygon selection tool and calculated in square micrometers (μm^2^). At least 10 muscle fibers were measured per sample to ensure statistical reliability.

### 2.3. Measurement of Meat Tenderness-Related Traits

Meat quality evaluations, including physicochemical measurements (pH, shear force, cooking loss, and pressing loss), were limited to the gluteus muscle, which represents a commercially relevant skeletal muscle [23]. Cardiac and esophageal muscles were not subjected to these tests, as they are not conventional meat products. For each analysis, approximately 5 g of tissue was used, and all tests were performed in triplicate.

Muscle pH was measured 24 h postmortem after rigor resolution at 4 °C. A portable pH meter (PH5, Tianxiang Feiyu Technology Co., Ltd., Beijing, China) was inserted directly into the center of the muscle sample to obtain readings without homogenization or dilution [24].

Muscle shear force was determined using a texture analyzer (C-LM3B, Beijing Tianxiang Feiyu Technology Co., Ltd., Beijing, China), following the *Warner–Bratzler* shear force protocol recommended by the *American Meat Science Association* (AMSA, 2016). After aging for 7 days at 0–4 °C, cylindrical muscle samples (~1.5 cm diameter, 2.0 cm height, and ~5 g weight) were prepared. Prior to testing, all samples were equilibrated to room temperature (~20 °C). A Warner–Bratzler V-shaped blade was used to shear the samples at a constant crosshead speed of 200 mm/min. The shear force was applied perpendicularly to the fiber orientation. The shear force values are reported in kilograms-force (kg·f) [25].

Pressing loss was measured using a meat water-holding capacity analyzer (TenovoMeat-1, Tianxiang Feiyu Technology Co., Ltd., Beijing, China). Pressure was applied perpendicularly to the muscle fibers. The samples were sampled 24 h postmortem and weighed (W1). The sample was placed between 16 layers of filter paper on both sides and compressed between two hard plastic plates under a pressure of 68.66 kPa for 5 min. After compression, the sample was wiped dry and weighed again (W2). Pressing loss was calculated using the formula: pressing loss (%) = [(W1 − W2)/W1] × 100% [24].

Cooking loss was assessed by sealing samples in plastic cooking pouches and immersing them in a 75 °C water bath (GD120, Grant, Shepreth, UK) for 45 min until the internal temperature reached 70 ± 2 °C. After cooling to room temperature, samples were wiped and re-weighed. Cooking loss was calculated as the percentage of weight loss during cooking [26].

### 2.4. Whole-Genome Bisulfite Sequencing and Data Analysis

Muscle tissue samples were dissected within 30 min postmortem and immediately frozen in liquid nitrogen, followed by long-term storage at −80 °C until genomic DNA extraction. No additional stabilizing reagents were used, as the downstream analysis focused on DNA methylation. Whole-genome bisulfite sequencing (WGBS) libraries were constructed following the protocol recommended by the sequencing service provider (BGI-Shenzhen, China). Sequencing was performed on the MGISEQ-2000 platform, generating 150 bp paired-end (PE150) reads.

The raw sequencing data were subjected to quality control to remove reads containing adapter contamination, poly-N, or low-quality bases. Specifically, reads were filtered based on the following criteria: (i) reads with more than 10% unknown bases (N); (ii) reads containing more than 50% bases with Phred quality score ≤ 5; and (iii) adapter-contaminated reads. After filtering, high-quality clean reads were retained for downstream analysis.

The clean reads were aligned to the Bos taurus reference genome (GCF_000003205.7_Btau_5.0.1, downloaded from NCBI) using Bismark (version 0.22.3) [27], a specialized bisulfite aligner. Bismark was run in default mode, and only uniquely mapped reads were retained. Methylation calls for cytosines in the CG, CHG, and CHH contexts were extracted using Bismark’s methylation extractor module. The methylation level of each cytosine was calculated as the number of reads supporting methylated cytosines divided by the total number of reads covering the site.

Differentially methylated regions (DMRs) between MT and WT cattle were identified using the DSS (Dispersion Shrinkage for Sequencing data) R package. Regions were considered differentially methylated if they satisfied the criteria of a *p*-value < 0.05 and a minimum methylation fold change of ≥1.5. DMRs were annotated to nearby genes based on the Bos taurus reference genome annotation. KEGG pathway enrichment analysis of DMR-associated genes was performed using the clusterProfiler package (v4.6.2) in R [28].

### 2.5. Transcriptome Sequencing and Differential Expression Analysis of Muscle Tissues

Total RNA was isolated from skeletal muscle, heart muscle, and smooth muscle tissues using standard protocols, followed by integrity assessment to ensure high-quality RNA for library construction. Transcriptome libraries were generated and sequenced using the MGISEQ-2000 platform, producing paired-end reads of 150 bp in length.

Raw RNA-seq reads were aligned to the *Bos taurus* reference genome (GCF_000003205.7_Btau_5.0.1) using HISAT2 (v2.2.1) [29]. Transcript assembly and quantification were performed using StringTie (v2.2.1), and raw read counts were extracted using the prepDE.py script provided by the StringTie package. Differentially expressed genes (DEGs) between MT and WT groups were identified using DESeq2 (v1.38.3) with default parameters [30]. Genes with an adjusted *p*-value < 0.05 and |log₂ fold change| ≥ 1 were considered significantly differentially expressed. Ballgown (v2.30.0) was used only for transcript visualization and descriptive statistics of normalized expression values [31].

Principal component analysis (PCA) was performed based on log₂-transformed fragments per kilobase of transcript per million mapped reads (FPKM) values. Genes with zero expression across all samples were removed. The PCA was conducted using the prcomp() function in R (v4.6.2), with centering and scaling enabled. The proportion of variance (PV) and standard deviation (SD) for each principal component were computed from the eigenvalues. PCA visualization was carried out using the ggplot2 packages to assess overall expression divergence among muscle types and genotypes. KEGG pathway enrichment analysis of the DEGs was performed using the *clusterProfiler* R package (v4.6.2) to explore the signaling pathways involved in gene expression differences across skeletal, cardiac, and smooth muscle tissues.

### 2.6. Data Statistical Analysis

Statistical analyses of muscle tenderness indicators (including pH, shear force, pressing loss rate, and cooking loss rate) and muscle area measurements were conducted using GraphPad Prism software (version 8.3.0, GraphPad Software, San Diego, CA, USA). DEGs identified by DESeq2 were visualized using GraphPad Prism. Differences between two muscle groups (MT vs. WT) were analyzed using unpaired two-tailed Student’s *t*-tests. Results are presented as mean ± standard deviation (SD). Each group consisted of at least three biological replicates. *p* < 0.05 was considered statistically significant.

## 3. Results

### 3.1. Differences in Morphology Across Three Muscle Types Between MT and WT Cattle

**Figure 2:**
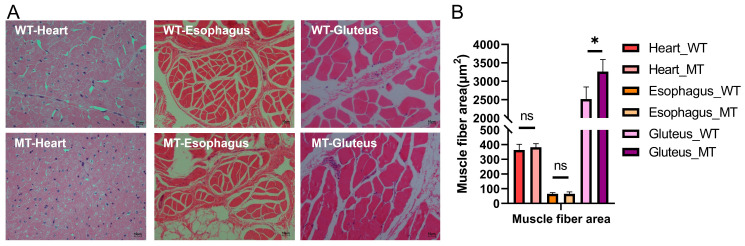
Histological morphology and muscle fiber area analysis of heart muscle, esophageal muscle, and gluteus muscle in MT and WT cattle. (**A**) Representative HE-stained sections showing tissue morphology of the three muscle types. (**B**) Quantification of muscle fiber area for each muscle type. *p* < 0.05 was considered statistically significant. * *p* < 0.05; ns, not significant.

The three selected muscles tissue—heart, esophagus (smooth muscle), and gluteus (skeletal muscle)—exhibited distinct morphological structures. Histologically, the heart muscle displayed densely packed and uniformly arranged fibers with narrow intercellular spaces, characteristic of highly oxidative tissue. The esophagus muscle featured a looser fiber arrangement, abundant connective tissue, and a relatively coarse fiber structure. In contrast, the gluteus muscle showed a more typical skeletal muscle architecture with relatively thick and aligned fibers, indicating its primary role in locomotion. Compared to WT cattle, MT cattle exhibited enlarged muscle fiber size in the gluteus muscle. However, no significant differences were observed in the heart and esophageal muscles between the two groups (Figure 2A). Quantification of muscle fiber area (Figure 2B) revealed a significant increase in the gluteus muscle of MT cattle compared to WT cattle (3263.06 ± 327.30 vs. 2512.56 ± 332.52 μm^2^, *p* = 0.049). In the esophageal smooth muscle, where fibers are spindle-shaped and variably oriented, we specifically assessed the cross-sectional area of smooth muscle cells. No significant differences were detected in muscle fiber area in the heart (382.30 ± 24.36 vs. 363.94 ± 38.29, *p* > 0.05) and esophagus muscles (65.69 ± 13.58 vs. 65.54 ± 8.78, *p* > 0.05) between the two groups.

These morphological differences were reflected in the physical and chemical properties of the gluteus muscle (Table 1). In terms of tenderness, measured by shear force, the gluteus muscle exhibited significantly lower shear force in MT cattle compared to WT (MT: 1.743 ± 0.460 kg·f; WT: 2.424 ± 0.443 kg·f; *p* = 0.044), indicating improved tenderness. The gluteus muscle also showed a lower cooking loss rate in MT cattle (MT: 30.99 ± 1.296%; WT: 35.66 ± 2.106, *p* = 0.0029), along with a significant decrease in pH in the MT group (*p* = 0.0143).

Taken together, these data demonstrate that muscle-specific morphological traits are closely associated with differences in meat quality parameters, and that *MSTN* knockout may exert muscle-type-dependent effects on postmortem pH, water retention, and tenderness.

### 3.2. Comparative Analysis of DNA Methylation and Gene Expression in Different Muscle Types of Cattle

To further explore the relationship between methylation changes and transcriptional regulation, genome-wide DNA methylation profiles and RNA-seq data were analyzed in the heart, esophagus, and gluteus tissues from MT and WT cattle.

**Figure 3:**
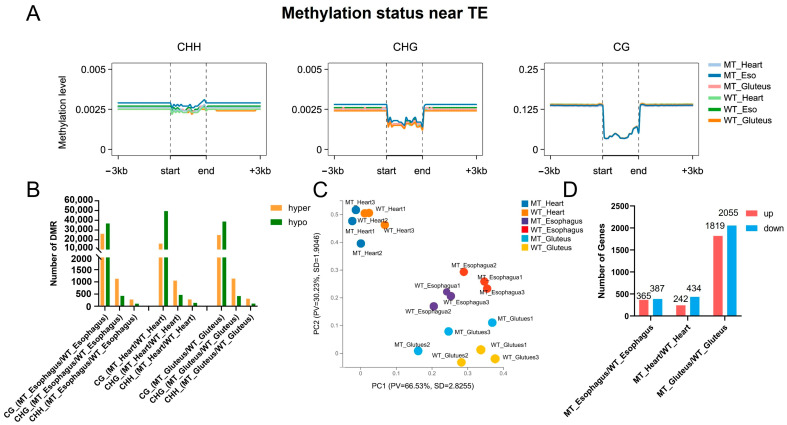
DNA methylation status and gene expression profiles in three muscle types of cattle. (**A**) DNA methylation levels near transposable elements (TEs) across skeletal muscle (gluteus), heart muscle, and smooth muscle (esophagus). (**B**) Number of differentially methylated regions (DMRs) identified among the three muscle types. (**C**) Principal component analysis (PCA) of transcriptomic profiles showing clustering patterns of skeletal muscle, heart, and smooth muscle. (**D**) Expression patterns of differentially expressed genes (DEGs) among the three muscle types.

The cytosine coverage statistics show that the majority of cytosines in all tissues and groups (MT and WT) had relatively low sequencing depth (0–10×), with over 75% of sites falling into this range, while only a small proportion of sites had high coverage (>30×), indicating an overall moderate sequencing depth across samples (Supplementary Table S3). The methylation landscape of CG, CHG, and CHH contexts exhibited distinct distribution patterns across the three muscle types (heart, esophagus, and gluteus). CG methylation showed the highest levels (98.06–99.00%, Supplementary Table S4) among the three contexts, with a sharp decrease near the transcription start site (TSS), whereas CHG and CHH methylation levels remained relatively low and stable across genomic regions (Figure 3A).

Differentially methylated regions (DMRs) were identified between MT and WT groups, with CG context displaying the highest number of DMRs across all muscle types (Figure 3B). The gluteus muscle exhibited the greatest number of DMRs in all three muscles, followed by the heart and esophagus (Supplementary Table S4). In all cases, hypermethylation events were more prevalent than hypomethylation, suggesting a potential regulatory role in gene expression. This distribution also mirrors the morphological and meat quality results, where the gluteus muscle showed the most pronounced differences between MT and WT cattle, further highlighting its sensitivity to *MSTN* gene editing.

Transcriptome profiling via principal component analysis (PCA) revealed distinct clustering of tissue types, with MT and WT gluteus samples exhibiting clear separation, suggesting significant transcriptional reprogramming in muscle tissue. In contrast, heart and esophagus samples showed smaller genotype-driven variation. Notably, the heart samples clustered farther from the gluteus and esophagus tissues, indicating greater divergence in gene regulatory programs and expression profiles between cardiac muscle and the other two muscle types (Figure 3C). Consistently, differential expression analysis revealed the greatest number of differentially expressed genes (DEGs) in gluteus tissue, with 2055 genes downregulated and 1819 upregulated in MT compared to WT. Fewer DEGs were identified in the esophagus (365 upregulated and 387 downregulated) and heart (242 upregulated and 434 downregulated, Figure 3D), suggesting that *MSTN* deletion exerts a more pronounced transcriptional impact on skeletal muscle than on other muscle-rich tissues.

### 3.3. Comparative Functional Transcriptomic Analysis Across Muscle Types

**Figure 4:**
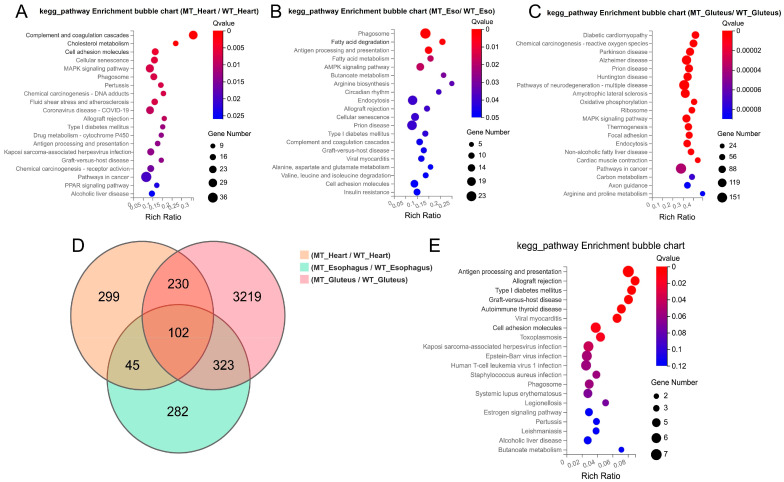
Functional enrichment analysis of differentially expressed genes (DEGs) among the three muscle types. (**A**–**C**) KEGG pathway bubble plots of DEGs in heart, smooth muscle, and skeletal muscle, respectively. (**D**) Venn diagram illustrating the overlap of DEGs among the three muscle types. (**E**) KEGG enrichment bubble plot of 102 common DEGs shared across all three muscle types.

KEGG pathway enrichment analysis revealed distinct functional differences among the three muscle types between MT and WT cattle. In the heart muscle, differentially expressed genes (DEGs) were significantly enriched in pathways related to complement and coagulation cascades, cholesterol metabolism, cell adhesion molecules, MAPK signaling pathway, and antigen processing and presentation (Figure 4A). In the esophageal muscle, significant enrichment was observed in pathways associated with phagosome formation, fatty acid degradation, AMPK signaling, butanoate metabolism, arginine biosynthesis, and circadian rhythm. Additionally, immune-related pathways, such as allograft rejection, and antigen processing and presentation, were also enriched (Figure 4B). In the gluteus muscle, DEGs were predominantly enriched in pathways related to oxidative phosphorylation, carbon metabolism, axon guidance, and muscle contraction. Notably, several pathways associated with neurodegenerative diseases, including Alzheimer’s disease, Parkinson’s disease, and Huntington’s disease, were significantly enriched (Figure 4C).

A Venn diagram analysis (Figure 4D) revealed a total of 102 DEGs commonly shared across the three muscle types. KEGG analysis of these shared genes (Figure 4E) demonstrated significant enrichment in antigen processing and presentation, type I diabetes mellitus, cell adhesion molecules, and various viral infection and autoimmune-related pathways, indicating that *MSTN* gene-editing consistently affects immune and stress-response mechanisms in muscle tissues. Taken together, these results demonstrate that *MSTN* gene editing leads to muscle hypertrophy and alters key physiological and molecular features in a muscle type-specific manner, with shared transcriptional reprogramming involving immune regulation and metabolic remodeling.

### 3.4. Comparative Functional Analysis of DNA Methylation Patterns Among Muscle Types

**Figure 5:**
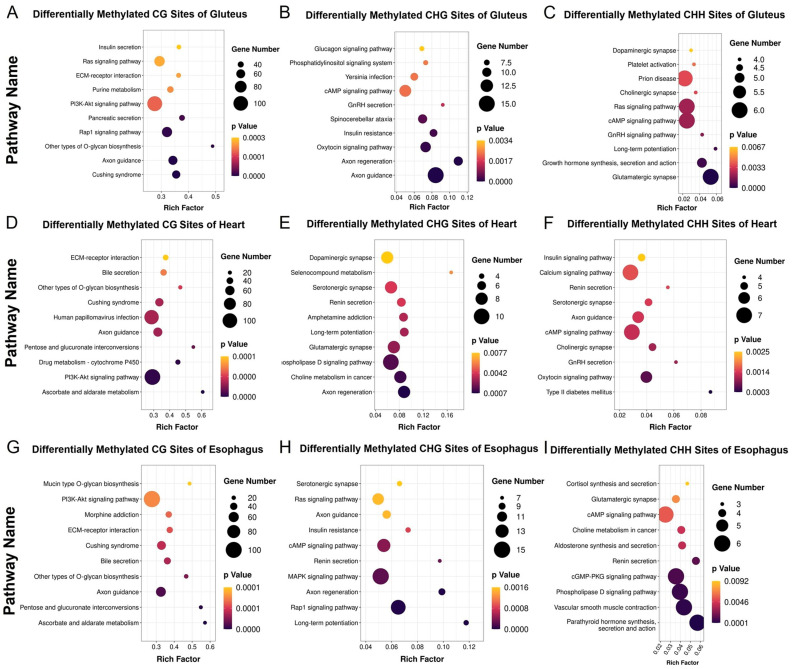
KEGG enrichment analysis of differentially methylated CH, CHG, and CHH sites between MT and WT cattle. (**A**–**C**) KEGG pathway enrichment of differentially methylated cytosines (mCH, mCHG, and mCHH) in skeletal muscle. (**D**–**F**) KEGG pathway enrichment of mCH, mCHG, and mCHH in heart muscle. (**G**–**I**) KEGG pathway enrichment of mCH, mCHG, and mCHH in smooth muscle.

In the gluteus muscle, differentially methylated sites (DMSs) in all three methylation contexts (CHH, CHG, and CG) were significantly enriched in axon guidance and axon regeneration, suggesting epigenetic regulation of neuronal signaling and muscle remodeling. Other enriched pathways included dopaminergic synapse, cholinergic synapse, insulin signaling, and PI3K-Akt signaling, indicating potential influences on neuromuscular function and metabolic regulation (Figure 5A–C). Consistent with previous gene expression analysis, KEGG enrichment of gluteus DEGs revealed pathways related to axon guidance, muscle contraction, and neurodegenerative diseases. These transcriptomic changes, aligned with methylation patterns, suggest coordinated regulation of neuronal and metabolic processes in MT cattle muscle.

In the heart muscle, DMSs were significantly enriched in axon guidance, as well as cAMP signaling, serotonergic synapse, and calcium signaling pathways (Figure 5D–F). These findings suggest that DNA methylation may epigenetically regulate cardiac contractility and intracellular signal transduction. Correspondingly, DEGs in the heart were enriched in pathways such as cell adhesion molecules and MAPK signaling, indicating potential transcriptional modulation.

In the esophagus (smooth muscle), DMSs across all three methylation contexts were consistently enriched in the axon guidance pathway, as well as in the PI3K-Akt signaling pathway, ECM–receptor interactions, and glycosaminoglycan biosynthesis. These findings suggest that *MSTN* gene editing may influence extracellular matrix remodeling and neuromuscular regulation in smooth muscle through DNA methylation. In line with this, the previously identified DEGs in smooth muscle were significantly enriched in pathways related to phagosome formation, fatty acid degradation, and AMPK signaling—metabolic and immune-associated processes (Figure 5G–I).

### 3.5. Differential Expression of Axon Guidance-Related Genes Across Muscle Types

**Figure 6:**
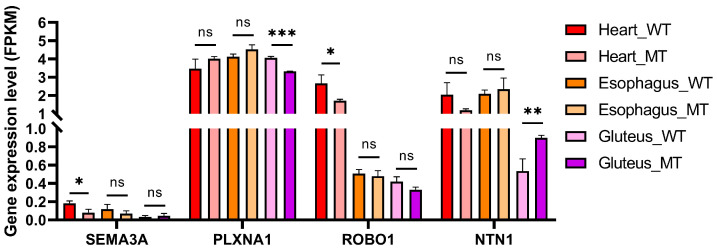
Expression levels of axon guidance-related genes in three muscle types of WT and MT cattle. FPKM values of *SEMA3A*, *PLXNA1*, *ROBO1*, and *NTN1* were compared between WT and MT cattle in heart, esophageal, and gluteus muscles. Data are presented as mean ± SD (*n* = 3 per group). * *p* < 0.05, ** *p* < 0.01, and *** *p* < 0.001; ns, not significant.

To further explore *MSTN*-dependent transcriptional regulation of the axon guidance pathway across different muscle types, we utilized RNA-Seq data obtained from gluteus, heart, and esophageal muscles to quantify the expression levels of four representative axon guidance-related key genes, *SEMA3A*, *PLXNA1*, *ROBO1*, and *NTN1*, that were both significantly differentially expressed and functionally enriched in the axon guidance pathway (Figure 6).

The expression of *SEMA3A* was significantly decreased in the heart muscle of MT cattle compared with WT (*p* < 0.05), while no significant changes were observed in esophageal or gluteus muscles. *PLXNA1* exhibited a significant decrease only in gluteus muscle (*p* < 0.001), whereas its expression remained stable in the heart and esophagus across genotypes. *ROBO1* showed a downward trend in MT cattle in all muscle types, but without statistical significance.

Interestingly, *NTN1* expression was significantly upregulated in gluteus muscle of MT cattle (*p* < 0.01), while no changes were detected in heart and esophagus. These results suggest muscle-specific regulatory effects of myostatin deficiency on axon guidance gene expression, particularly in skeletal muscle.

## 4. Discussion

### 4.1. Morphology and Meat Quality of MSTN-Edited Cattle Muscles

Histological examination using HE staining revealed distinct fiber morphology among skeletal, cardiac, and smooth muscles. In particular, the cross-sectional area of muscle fibers was significantly increased in MT cattle, most prominently in skeletal muscle. This finding is in line with *MSTN*’s established role as a negative regulator of myogenesis. *MSTN* signals through activin type IIB receptors, activating SMAD2/3 transcription factors, which suppress myoblast proliferation and differentiation [13,32]. Disruption of *MSTN* removes this inhibitory pathway, thereby promoting muscle fiber hypertrophy through enhanced satellite cell activity and protein synthesis [33].

Compared to WT cattle, MT cattle demonstrated significantly improved tenderness and reduced cooking loss in the gluteus muscle. These changes are likely associated with hypertrophic muscle fiber growth and altered extracellular matrix remodeling following *MSTN* disruption: Myostatin normally inhibits satellite cell proliferation and regulates connective tissue deposition [34]. Thus, its deletion may enhance muscle fiber diameter and affect collagen content, potentially contributing to improved tenderness and water-holding capacity. Although we did not directly quantify intramuscular collagen or connective tissue density in this study, previous studies have reported reduced perimysial collagen content and lower collagen cross-linking in *MSTN*-deficient animals [35]. These findings support the hypothesis that lower shear force values observed in *MSTN*-KO cattle may be partially attributed to such structural changes. Further investigation using histological or biochemical collagen assays is warranted to validate this speculation.

In summary, these findings suggest that *MSTN* editing exerts measurable effects on muscle morphology and skeletal muscle meat-quality parameters.

### 4.2. DNA Methylation and Transcriptional Modulation of Muscle Function

In addition to phenotypic changes in muscle morphology, it is important to understand the underlying molecular mechanisms. DNA methylation is a key epigenetic modification regulating gene transcription and is known to affect muscle development, metabolism, and differentiation [36,37].

The axon guidance pathway, although traditionally associated with neural development, has been increasingly recognized for its role in muscle fiber formation, neuromuscular junction establishment, and cell–cell communication within muscle tissue. These changes were especially prominent in skeletal muscle, consistent with its higher plasticity and relevance to meat production [38,39]. Mechanistically, this may be explained by the overall lower DNA methylation level observed in skeletal muscle, particularly in promoter regions. Such hypomethylation is known to support a more open chromatin state and enhanced transcriptional activity [40]. These regulatory layers may contribute to the activation of growth-related pathways, increased protein synthesis, and improved muscle quality traits in *MSTN*-KO animals. Although post-translational mechanisms were not directly assessed, their role in fine-tuning these processes cannot be excluded and warrants future study.

Interestingly, recent studies have shown that *MSTN* also plays important roles in axon guidance signaling. MSTN and its homolog GDF11 are expressed in neurons and have been reported to affect synapse formation and neuronal morphology [41]. For example, in Drosophila, the MSTN homolog Myoglianin (MYO) regulates synaptic strength and composition at the neuromuscular junction via a Smad2-dependent mechanism. Moreover, MSTN and GDF11 were found to inhibit neurite outgrowth and synaptogenesis in cultured rat cortical neurons, suggesting a conserved role in neurodevelopmental regulation [42]. These findings support the hypothesis that epigenetic and transcriptional modulation of axon guidance genes may influence muscle development, potentially contributing to observed differences in muscle structure. Several axon guidance molecules, such as *SEMA3A*, *NTN1*, and *EPH/ephrin* families, have been shown to regulate myoblast adhesion, differentiation, and muscle innervation [43,44,45]. However, the direct impact of these molecular changes on meat quality traits such as tenderness or cooking loss remains to be further investigated.

The enrichment of cAMP and MAPK-related pathways in cardiac tissue suggests the presence of a signaling cascade analogous to the cAMP/PKA–MAPK axis, known for its role in synaptic plasticity in neurons. PKA may inhibit Ras activity, indicating competitive regulation between PKA- and Ras-mediated MAPK activation [46,47]. This convergence may influence cardiac plasticity and contractile function. In the esophagus, coordinated changes in methylation and expression implicate *MSTN* in metabolic adaptation and immune regulation, potentially via AMPK/NOX4/PI3K/AKT signaling, which has established links to glucose homeostasis and insulin resistance [48,49].

To further investigate this connection, we analyzed the expression profiles of four representative axon guidance-related genes—*SEMA3A*, *PLXNA1*, *ROBO1*, and *NTN1*—across heart, esophageal, and gluteus muscles. Notably, *SEMA3A*, a well-characterized inhibitory cue for axonal pathfinding and muscle fiber outgrowth [50], was significantly downregulated in the heart of MT cattle. This downregulation may reflect a disruption of *MSTN*-dependent regulatory pathways. MSTN, as a member of the TGF-β superfamily, signals primarily through SMAD2/3-mediated transcriptional mechanisms. Previous studies have demonstrated that *SEMA3A* transcription is positively regulated by TGF-β signaling via SMAD binding elements in its promoter region [51,52]. Therefore, the loss of *MSTN* could lead to decreased SMAD2/3 activation, reducing transcriptional activation of *SEMA3A*. This reduced expression of *SEMA3A* could relieve its inhibitory effect on cardiomyocyte outgrowth and cytoskeletal remodeling, thereby contributing to tissue-specific adaptations in muscle structure or performance. Interestingly, both *ROBO1* and *SEMA3A* were upregulated in the cardiac muscle of MT cattle, suggesting a coordinated regulatory mechanism. This pattern is consistent with previous findings that mutations in axon guidance-related genes such as *ROBO1* and *SEMA3A* are often associated with congenital heart defects and diaphragm malformations, implying that these genes play critical roles in cardiac morphogenesis and structural integrity [53,54]. *PLXNA1*, a receptor for *SEMA3A*, exhibited relatively stable expression across most tissues but was significantly downregulated in the gluteus muscle of MT cattle. Although the precise significance of *PLXNA1* and *SEMA3A* expression patterns across different muscle types remains unclear, the observed gluteus-specific suppression of *PLXNA1* may attenuate *SEMA3A*-mediated repulsive signaling, potentially reducing inhibitory cues and thereby creating a more permissive environment for axonal extension or myofiber growth [55]. Importantly, *NTN1* (netrin-1), a guidance molecule known for its dual role in mediating both attractive and repulsive signaling, is significantly upregulated in the gluteal muscle of MT cattle. This suggests a potential role for *NTN1* in promoting muscle innervation and maintaining fiber integrity. Notably, this alteration has been associated with intramuscular fat deposition in pigs, indicating that *NTN1* may synergize with *MSTN* to regulate both adipogenesis and muscle development [56,57]. These expression changes support the hypothesis that *MSTN* deletion induces a coordinated regulation of axon guidance genes, contributing to improved muscle innervation, fiber integrity, and enhanced muscle structure and function.

We acknowledge that the current study is limited by the relatively small sample size, which is mainly due to the challenges in producing and maintaining *MSTN* gene-edited cattle with consistent genetic backgrounds. In addition, the study lacks direct functional validation of these genes in muscle development. The relationships between altered axon guidance gene expression and muscle phenotype remain correlative, and the precise mechanisms through which these molecular changes influence myofiber formation, fiber type composition, or innervation remain to be elucidated. Additionally, while the integrative transcriptomic and epigenetic analyses provide strong indirect support, experimental validation using loss- or gain-of-function assays in cellular or animal models would be essential to confirm the causal roles of these genes and pathways.

## 5. Conclusions

Our findings reveal that *MSTN* gene editing in cattle enhances skeletal muscle structure and tenderness, as evidenced by physicochemical analyses performed on the gluteus muscle. These results indicate improvements in specific physical characteristics of skeletal muscle but do not encompass sensory, commercial, or consumer-based measures of meat quality. Additionally, *MSTN* disruption induced distinct transcriptional and epigenetic alterations in cardiac and smooth muscles. The identification of axon guidance as a shared regulatory axis underscores the broader role of *MSTN* in muscle system regulation beyond skeletal muscle growth.

## Figures and Tables

**Table 1:** Comparison of gluteus muscle tenderness parameters in MT and WT cattle.

Indicator	MT	WT	*p*-Value
Shear force, SF (kg·f)	1.743 ± 0.46	2.424 ± 0.443	0.044118
Extrusion loss rate (%)	5.406 ± 1.028	4.886 ± 0.765	0.390872
Cooking loss rate (%)	30.99 ± 1.296	35.66 ± 2.106	0.002877
pH	5.316 ± 0.09	5.634 ± 0.21	0.014338

## Data Availability

The original contributions presented in the study are included in the article/Supplementary Materials; further inquiries can be directed to the corresponding author.

## Supplementary Materials

The following supporting information can be downloaded at https://www.mdpi.com/article/10.3390/cimb47060431/s1.
