# Supplementary material for: Loss of Myostatin Shapes the Transcriptomic and Epigenetic Landscapes Across Multiple Muscle Types in Cattle
Source: Curr Issues Mol Biol. 2025 Jun 7;47(6):431. doi: 10.3390/cimb47060431 (PMC12192037; doi:10.3390/cimb47060431)
Supplement: Supplementary file 1 [file cimb-47-00431-s001.zip › Supplementary Materials.pdf]

Table S1. Feed composition

Feed composition	Quality(kg)/day
Silage (kg/head)	12
Gluten (kg/head)	2
Hay (bale/head)	2
Refined feed	2.5

Table S2. Nutrient composition of refined feed

Nutrients	Composition
Crude protein, not less than	16.0
Crude fat, not more than	12.0
Crude fiber, not more than	9.0
Calcium	0.5-1.8
Total phosphorus, not less than	0.4
Sodium chloride	0.8-1.5
Lysine, not less than	0.4

Table S3. Summary of cytosine coverage statistics.

**Coverage**	**MT-Heart**	**WT-Heart**	**MT-Esophagus**	**WT-Esophagus**	**MT-Gluteus**	**WT-Gluteus**
0-5	43.72	43.07	39.16	40.99	42.11	38.56
5-10	34.83	33.64	35.69	36.18	35.81	37.23
10-20	20.17	21.6	23.43	21.62	20.83	22.89
20-30	1.04	1.44	1.48	0.99	1.03	1.09
30-40	0.06	0.07	0.07	0.05	0.06	0.03
40-50	0.03	0.03	0.03	0.03	0.03	0.03
50-60	0.02	0.02	0.02	0.02	0.02	0.02
60-70	0.02	0.02	0.02	0.02	0.01	0.02
70-80	0.01	0.01	0.01	0.01	0.01	0.01
80-90	0.01	0.01	0.01	0.01	0.01	0.01
90-100	0.01	0.01	0.01	0.01	0.01	0.01
>100	0.09	0.09	0.09	0.09	0.09	0.1

Table S4. Summary of cytosine methylation types (mCG, mCHG, and mCHH) in three muscle types of cattle.

Sample	C	CHG	CG	CHH
MT_Heart	29497499	129226 (0.438%)	28996300 (98.30%)	371973 (1.26%)
WT_Heart	30653332	76562 (0.250%)	30345339 (99.00%)	231431 (0.75%)
MT_Gluteus	30383188	133336 (0.439%)	29850260 (98.25%)	399592 (1.32%)
WT_Gluteus	32247704	80055 (0.248%)	31902829 (98.93%)	264820 (0.821%)
MT_Esophagus	29994709	136602 (0.455%)	29413046 (98.06%)	445061 (1.48%)
WT_Esophagus	30188431	78078 (0.259%)	29838090 (98.84%)	272263 (0.902%)
